# Diagnosis of knee joint invasion in patients with osteosarcoma: the value of direct and indirect MRI findings

**DOI:** 10.55730/1300-0144.6009

**Published:** 2025-06-10

**Authors:** İpek TAMSEL, Hüseyin KAYA, Orkhan AGHAMIRZAYEV, Oğuz DİMDORK, Başak DOĞANAVŞARGİL, Mehmet ARGIN, Dündar SABAH

**Affiliations:** 1 Department of Radiology, Faculty of Medicine, Ege University, İzmir, Turkiye; 2 Department of Orthopedics and Traumatology Tumor Surgery, Faculty of Medicine, Ege University, İzmir, Turkiye; 3 Department of Pathology, Faculty of Medicine, Ege University, İzmir, Turkiye; 4 Hand Microsurgery Orthopaedics and Traumatology (EMOT) Hospital, İzmir, Turkiye

**Keywords:** Bone neoplasms, neoplasm staging, knee, osteosarcoma, magnetic resonance imaging

## Abstract

**Background/aim:**

Osteosarcoma is the most common primary malignant bone tumor in adolescents, and the evaluation of joint invasion with MRI is important for treatment planning. This study aimed to investigate the diagnostic value of MRI findings (direct and indirect) for joint invasion in patients diagnosed with osteosarcoma of the knee region.

**Materials and methods:**

The MRI evaluations of 50 knee osteosarcoma patients who underwent surgical resection between 2006 and 2018 were reviewed retrospectively by two radiologists and an orthopedic oncologist. The presence of intrasynovial tumor tissue, intra-articular destruction of cartilage or bone, and invasion of the capsular and cruciate ligament insertions were evaluated as direct findings in the diagnosis of joint invasion on MRI. Indirect findings included tumor size, adjacent epiphyseal bone signal changes- bone marrow infiltration and edema, synovial contrast enhancement, and joint effusion. These findings were scored separately on a 5-point Likert scale and statistically compared with histopathologic results

**Results:**

The mean age of the patients was 22 years and the gender distribution was 21 females and 29 males. The best predictors for joint invasion were direct visualization of capsular insertion invasion (p < 0.05) and destruction of intraarticular bone (p < 0.05). MRI findings with statistically significant sensitivity and specificity: intrasynovial tumor tissue specificity 76%, sensitivity 58%; intra-articular cartilage destruction specificity 84%, sensitivity 56%; intra-articular bone destruction sensitivity 84%, specificity 48%; capsular insertion invasion sensitivity 92%, specificity 48%. Synovial effusion and contrast enhancement were the most sensitive indirect signs but lacked specificity.

**Conclusion:**

Joint invasion by osteosarcoma can reliably be assessed on preoperative MR images with high sensitivity and specificity. Particularly direct visualization of intrasynovial tumor tissue, capsular insertion invasion, and destruction of intraarticular bone and cartilage, a combination of highly specific direct signs was valuable, while indirect signs were less predictive and specific.

## 1. Introduction

Osteosarcoma, the most common primary malignant bone tumor in adolescents, occurs in 75% of cases within the 15 to 25 age group. Approximately half of all osteosarcomas affect the knee region, with the distal femur being the most common site. Secondary osteosarcomas occur in the elderly, usually as a result of malignant degeneration of Paget’s disease, bone infarction, post-radiotherapy, osteochondroma, or osteoblastoma [1–4].

Multimodality treatment including neoadjuvant/adjuvant chemotherapy, radiotherapy, and surgery has become the standard of care [5]. Surgical management and prognosis have improved with the advent of neoadjuvant chemotherapy. The favorable response of osteosarcoma to chemotherapy has led to more frequent use of limb-sparing surgery, resulting in better functional outcomes and survival rates [6]. The surgical approach is determined by the presence of joint invasion. While intra-articular resection can be performed in cases without joint invasion, extra-articular resection is required to prevent local recurrence in the presence of joint invasion [7,8]. The factors that determine whether the joint should be removed extra-articularly remain unclear in the literature. The type of surgery is based on clinical and radiological findings. During multidisciplinary team meetings, analysis of preoperative magnetic resonance imaging (MRI) is an important component of the decision-making process [3,4].

MRI is the standard imaging modality for local staging of malignant bone tumors [9,10]. By providing soft tissue contrast and three-dimensional anatomical information, extraosseous tumor growth and extension into the vascular-nerve bundle can be evaluated [11]. The evaluation of joint invasion in tumors adjacent to the joint using MRI is important for treatment planning. There are studies in the literature that demonstrate the feasibility of evaluating joint invasion by MRI in malignant bone tumors.

The presence of intrasynovial tumor tissue, intra-articular cartilage or bone destruction, and spread to the attachment sites of the capsule and ligaments are considered direct findings in the diagnosis of joint spread on MRI. These features represent direct tumor extension into the joint space, which is considered a critical criterion for surgical planning and prognosis. Destruction of cartilage and subchondral bone can be visualized as focal discontinuities or signal abnormalities in the normally hypointense cartilage layer on T2-weighted images

Indirect findings included tumor size, adjacent epiphyseal bone signal changes such as bone marrow infiltration and edema, synovial contrast enhancement, and joint effusion. These signs, although not specific, suggest reactive changes and increased likelihood of joint involvement. Bone marrow edema adjacent to the joint may reflect tumor-related inflammation or early infiltration. Joint effusion, although a nonspecific finding, is frequently associated with intra-articular processes and may accompany either direct invasion or reactive synovitis. Furthermore, a tumor size larger than 5 cm and proximity of the lesion to the articular surface have been shown to correlate with a higher risk of joint invasion, particularly in high-grade sarcomas. Therefore, both direct and indirect MRI findings must be interpreted together in a comprehensive diagnostic approach to assess joint invasion accurately [6,10,12,13].

This study aimed to investigate the diagnostic value of MRI features (direct and indirect) for joint invasion in patients with osteosarcoma of the knee region by analyzing them with histopathological results.

## 2. Methods

This study received ethical approval from the medical research ethics committee of the Ege University Faculty of Medicine, Medical Research Ethics Committee (approval number: 22 -7T/57, date: 29.07.2022). Informed consent was obtained from all patients before MRI, and no additional approval was necessary, as the study was conducted retrospectively.

**Figure 1:**
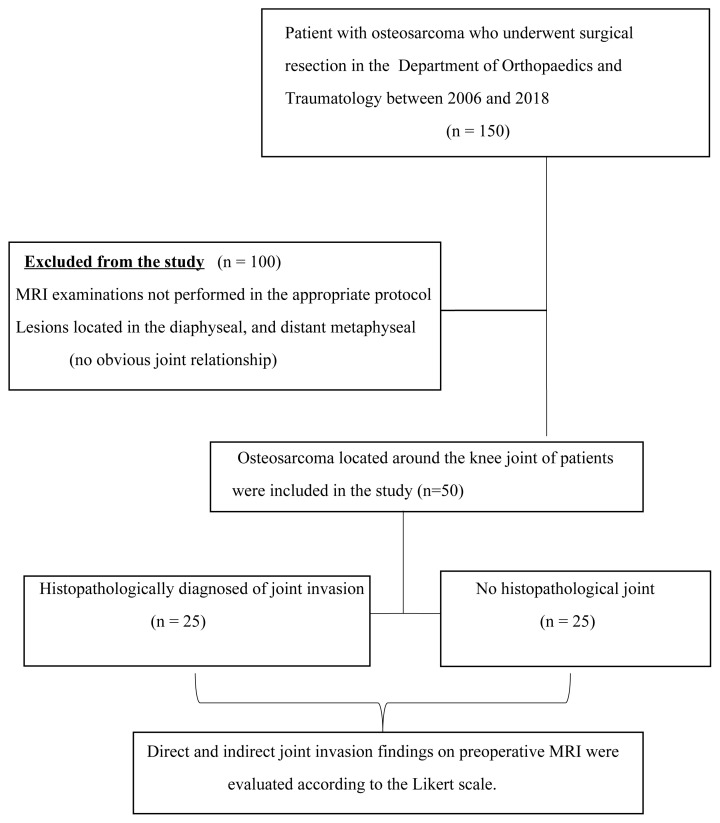
Flowchart of the study.

The flowchart of the study is summarized in Figure 1.

MR examinations of 50 osteosarcoma patients who underwent surgical resection in the Department of Orthopaedics and Traumatology, Division of Tumor Surgery, at Ege University Medical Faculty Hospital between 2006 and 2018 were retrospectively evaluated. Osteosarcomas located near the knee joint were included in our study. Cases with metaphyseal-diaphyseal localization not directly related to the joint were excluded from the study. The examinations of the patients were obtained from the imaging database. MRI scans compromised by artifacts or patient-related factors that prevented adherence to the standard imaging protocol were excluded from the study. In patients who received neoadjuvant chemotherapy, pre-treatment examinations were evaluated because neoadjuvant chemotherapy may cause a decrease in tumor size. However, in 5 patients in whom chemotherapy was not effective, posttreatment examinations were included in the study due to an increase in tumor size and were assessed for joint invasion.

MR examinations include images obtained using the tumor protocol in the Turkish Society of Radiology MR examination guidelines. In our study, images obtained from two MR devices (Amira and Magnetom Symphony, Siemens, Germany) producing a magnetic field strength of 1.5 Tesla were evaluated. In the MR examination, images were evaluated and obtained in at least two planes using T1W turbo spin echo (TR/TE: 611/11; matrix size: 70 × 320; NEX:1; slice thickness of 5 mm), fat-suppressed T2W turbo spin echo (TR/TE: 4670/12; matrix size: 70 × 320; NEX: 1; slice thickness of 5 mm), or Short tau inversion recovery (STIR) (TR/TE: 3000/40; matrix size: 70 × 320; NEX: 2; slice thickness of 5 mm) sequences. After gadolinium diethylenetriamine penta-acetic (Gd-DTPA) administration, images were obtained in dynamic and static fat-suppressed T1W sequences (at least in two planes). MR examinations with poor image quality caused by motion artifacts were excluded.

The time between the MRI examination and the operation is no more than 15 days. Two radiologists and an orthopedist experienced in tumor surgery (5 years) evaluated MR examinations using picture archiving and communication system (PACS) workstations. The evaluations were performed by two radiologists with experience in musculoskeletal imaging (9 and 15 years). The readers were blinded to histopathologic results.

In this study, direct and indirect MRI findings for the diagnosis of joint invasion were determined based on previous studies in the literature [6,9]. In the diagnosis of joint invasion, intrasynovial tumor tissue (tumor tissue definitively crossing the synovial membrane toward the joint space, having direct contact with synovial fluid), intra-articular destruction of cartilage or bone, capsular and cruciate ligament insertions invasion were evaluated as direct signs. For indirect signs, tumor size (tumor size was measured on the longest axis and expressed in millimeters), adjacent epiphyseal bone signal changes-bone marrow infiltration (iso-/hypointense to muscle tissue on T1W images), and edema (hypointense on T1W images, markedly hyperintense on fluid sensitive sequences), synovial contrast enhancement, joint effusion findings were evaluated. Discontinuity of intra-articular cortical bone and cartilage tissue (T1W and fluid-sensitive sequences) is defined as a sign of intra-articular destruction. Synovial effusion and synovial contrast enhancement were scored separately. Synovial enhancement characterized by thick synovium (>2 mm) was assessed on static postcontrast images.

Direct and indirect joint invasion findings were scored on a 5-point Likert scale with consensus among the readers. Joint invasion findings were evaluated on a 5-point Likert scale (1: invasion definitely present, 2: invasion probably present, 3: uncertain, 4: invasion probably absent, 5: invasion definitely absent) according to the consensus of three evaluators. Post-scoring evaluations were compared with pathology results. Only 2 patients scored 3 on the Likert scale, so we considered the MRI findings as invasion present/absent. The scores of all MRI findings were analyzed as an invasion (score 1–2)/no invasion (score 4–5).

### 2.1. Surgical technique

In cases of distal femur and proximal tibia osteosarcoma, intra-articular resection is performed if there is no knee joint invasion, while extra-articular resection is performed if there is knee joint invasion, as evaluated by the multidisciplinary tumor council.

In intra-articular resections, wide resection is done while preserving the patella and extensor mechanism, whereas, in extra-articular resections, subcutaneous flaps are created to expose the knee’s anterior surface, with resection including tissues related to the knee joint. Generally, femoral and tibial osteotomies are performed at least 2 cm from the tumor, based on preoperative MRI. After resection, the operation is completed with a tumor resection prosthesis and, additionally, the reconstruction of the extensor mechanism using a medial gastrocnemius muscle flap in cases where extra-articular resection was performed.

### 2.2. Histopathological evaluation

Tumor sections were preferably made in the sagittal plane. Coronal sections were made based on the size of the tumor mass and on the images recorded from radiological scans or findings previously noted on the preoperative tumor board. The section showing the largest extent of the tumor and best depicting the soft tissue and joint extensions was selected for ‘mapped sampling’. In all cases, samples containing tumor cortex relationship, tumor epiphysis relationship, and tumor joint relationship were separately taken, and where necessary, samples were also taken specifically for tibiofemoral joint or patellofemoral joint relationships. A median of 38 blocks ± 18 (Range: 15–95 blocks) was examined per case. Microscopic joint invasion was defined as either direct invasion of the joint cartilage, invasion of the synovium, or involvement of the joint capsule via soft tissue extension of the tumor. Histopathological evaluation results were accepted as the gold standard in terms of invasion.

### 2.3. Statistical analysis

All statistical analyses were performed using R software (R software, version 4.0.5, packages: arsenal, DTComPair, epiR, pROC, ggplot2. R Foundation for Statistical Computing, Vienna, Austria), and statistical significance was assessed at p < 0.05 in the study. For descriptive statistics, categorical variables were presented as frequencies and percentages, whereas numerical variables were reported as median and range (minimum-maximum).

The specificity, sensitivity, positive predictive value, and negative predictive value of the MRI findings were reported along with exact binomial 95% confidence intervals (CI). The agreement between MRI findings and pathological outcome (invasion present/absent) was evaluated using Percent Agreement (PA), Cohen’s/Conger’s κ, and Gwet’s AC coefficients. All coefficients were presented with 95% CI. Due to the problems associated with the kappa coefficient, Gwet’s AC, which provides more consistent and reliable results, was preferred. However, following the published guidelines, the other two coefficients were also included. The interpretation of the coefficients was conducted using Gwet’s probabilistic method based on the Landis and Koch scale.

The association between variables MR findings that were measured on a 5-point Likert scale and the pathological outcome was analyzed by Fisher’s Exact Test for Count Data. Tumor size was compared between invasion and noninvasion groups using Mann Whitney U test. The discriminative ability of tumor size for the presence of invasion was determined by the receiver operating characteristic (ROC) curve and the area under the curve was given at 95%. Specificity, sensitivity, positive predictive value, and negative predictive value of MR findings are given with exact binomial confidence interval. The predictive value of MR findings for pathological invasion outcome was evaluated by logistic regression in which variables were selected by the forward elimination method.

## 3. Results

The study included 50 patients who underwent resection for osteosarcoma in the knee joint region. The localization of the masses was distal femur in 34 patients (68%) and proximal tibia in 16 patients (32%). The mean age of the patients was 22 (range 9–70), and the sexdistribution was 21 female and 29 male. Lesion sizes were measured between 40 mm and 188 mm in the long axis. Extra-articular resection was performed in 35 patients based on the tumor board’s assessment of joint invasion. Histopathological analysis of resected specimens confirmed joint invasion in 25 patients, while the remaining 25 patients showed no evidence of invasion.

**Figure 2:**
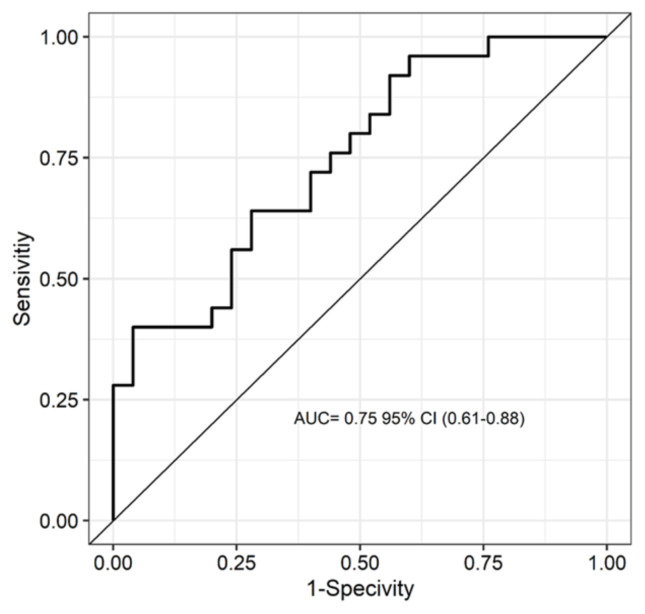
ROC analysis curves.

Comparative analysis revealed that the tumor size and volume were statistically significantly larger in the invasion group than in the no-invasion group (p = 0.002). The ability of tumor size to differentiate between invasive and noninvasive cases was evaluated using Receiver Operating Characteristic (ROC) analysis. The area under the curve (AUC) was calculated as 0.75 95% CI (0.61–0.88), indicating “acceptable” diagnostic performance. Details of the ROC analysis curve are presented in Figure 2.

MRI findings showed varying levels of sensitivity and specificity in identifying joint invasion (Table 1). The following MRI findings were statistically significant:

-Intrasynovial tumor tissue: Sensitivity 58%, specificity 76%.
-Intra-articular cartilage destruction: Sensitivity 56%, specificity 84%.
-Intra-articular bone destruction: Sensitivity 84%, specificity 48%.
-Capsular insertion invasion: Sensitivity 92%, specificity 48%.

When these MRI findings were examined together with logistic regression to predict the presence of invasion (present/absent versions), the presence of intra-articular cartilage destruction and the presence of capsular insertion invasion were the only significant findings in the model (R2 = 0.351, Hosmer Lemeshow p = 0.654, Model correct classification rate 73.5%), but the model could only correctly predict 58.3% of patients with invasion.

**Figure 3:**
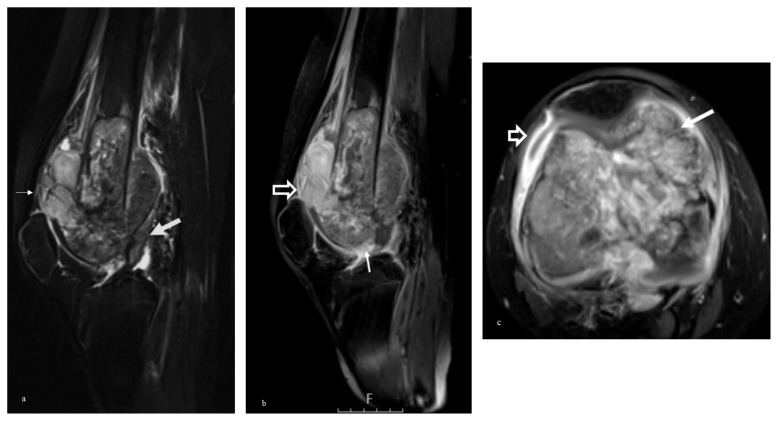
A 17-year-old man with osteosarcoma of the distal femur. Histopathologically, the joint invasion was diagnosed. This patient was assigned a score of 1 (definitely present) on the Likert scale due to the presence of joint capsule involvement, cruciate ligament infiltration, and intra-articular tumor tissue. The sagittal fat-suppressed T2 weighted image shows tumoral invasion in the joint capsule (arrow) and anterior cruciate ligament insertion (thick arrow) (a). Contrast-enhanced sagittal fat-suppressed T1 weighted image shows intrasynovial tumor tissue at suprapatellar joint recess (open arrow), intra-articular bone and cartilage destruction (arrow) at the intercondylar notch (b). Contrast-enhanced sagittal fat-suppressed T1 weighted image shows synovial enhancement at the anterior joint recess (open arrow) and intrasynovial tumor at the femoro-patellar joint compartment (arrow) (c).

**Figure 4:**
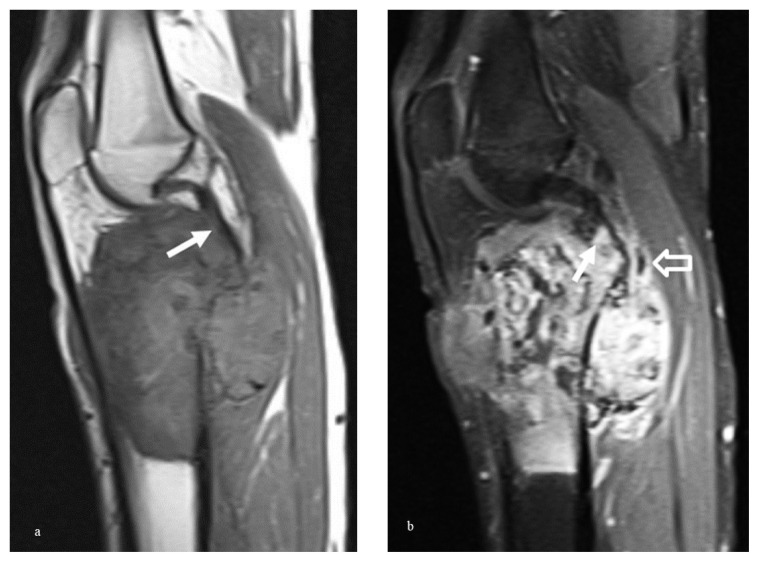
A 19 -year-old man with osteosarcoma of the proximal tibia. Histopathologically, the joint invasion was diagnosed. This patient was assigned a score of 1 (definitely present) on the Likert scale due to the presence of joint capsule involvement and posterior cruciate ligament infiltration. The sagittal T1-weighted image shows an inhomogeneous mass with soft tissue component at the proximal tibia, tumoral invasion at the posterior joint capsule, and posterior cruciate ligament insertion (arrow) (a). Corresponding contrast-enhanced sagittal fat-suppressed T1-weighted MR image shows heterojen enhancement at the tumoral mass, tumoral invasion of the posterior joint capsule (open arrow), and posterior cruciate ligament insertion (arrow) (b).

**Figure 5:**
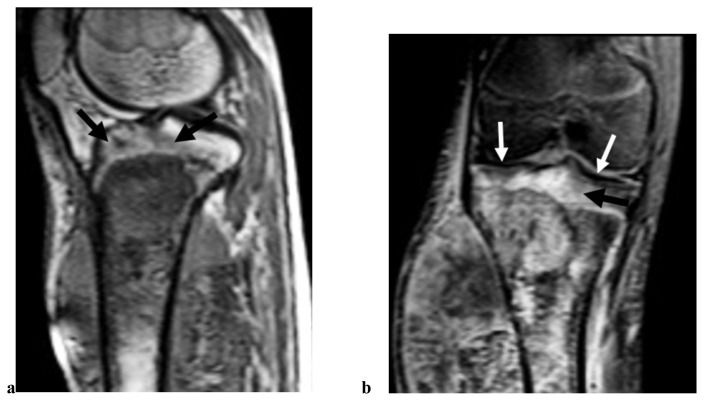
A 36-year-old male patient has osteosarcoma in the proximal tibia. Tumoral infiltration is observed in the epiphyseal region (Likert 1), while the cortical bone integrity is preserved in the subchondral area. No intrasynovial tumor or cruciate ligament infiltration was present (Likert 5). Postoperative histopathological finding of joint invasion was detected. The tumor appeared to invade the capsule at the lateral aspect of the joint. In the epiphyseal region, findings consistent with tumoral infiltration is observed, characterized by low signal intensity on sagittal T1W images (a) and contrast enhancement on coronal fat-suppressed T1W postcontrast image (black arrow) (b). Intra-articular cortical bone continuity (hypointense line) is preserved (arrow).

The relationship between specific MRI findings and histopathological evidence of invasion is detailed in Table 2. In diagnosing joint invasion, direct MRI observations of intrasynovial tumor tissue, intra-articular cartilage destruction, bone destruction, and capsular insertion invasion demonstrated high sensitivity. Representative MRI images illustrating these findings are provided in Figures 3a–3c and, Figures 4a, 4b. Conversely, indirect MRI findings, such as tumoral infiltrative signal changes within the epiphysis and epiphyseal edema, were not predictive of joint invasion, with p-values of 0.235 and 0.110, respectively (Figures 5a, 5b).

## 4. Discussion

This study evaluated the diagnostic performance of MRI features in detecting joint invasion in cases of osteosarcoma. Our patient series included patients with osteosarcoma localized to the knee region. Accurate preoperative assessment of joint invasion on MRI is of critical importance for orthopedic surgeons. False-positive diagnoses can lead to unnecessary wide resections, resulting in higher complication rates and poorer functional outcomes, while false-negative diagnoses increase the risk of local recurrence due to intraoperative dissemination of malignant cells.

The primary goal of surgical treatment for osteosarcoma is to remove the entire tumor with clear margins while preserving limb function whenever possible [13]. In the treatment of osteosarcoma with joint invasion, extra-articular excision or amputation is required to achieve tumor-free margins. The prevalence of joint involvement in studies reported in the literature has been noted to be between 19% and 24%. The invasion occurs through three pathways: beneath the joint capsule, at the bone-tendinous junction of intra-articular ligaments, and, particularly in cases with pathological fractures, by direct trans articular invasion [6,14]. In the knee joint, the endosteal capillaries within the cruciate ligaments are hypothesized to provide a direct anatomical pathway for tumor invasion. In contrast, avascular joint cartilage serves as a temporary barrier [15]. Contrast-enhanced MRI is reported to be 100% sensitive but only 69% specific in diagnosing joint invasion, with histopathological macroscopic and microscopic examination findings considered the reference gold standard [14]. Accurate assessment of joint invasion with preoperative MRI is important for the surgeon. False positive invasion diagnosis may lead to high complication rates and unnecessary wide excisions, while false negative diagnosis may increase the risk of early local recurrence due to inadequate resection.

The diagnostic performance of MRI features in detecting joint invasion was analyzed in comparison with histopathological findings. Tumor volume was higher in the group with invasion (p = 0.002). Previous studies have demonstrated that tumor size exceeding 5 cm and close proximity to the articular surface are significant risk factors for joint invasion, especially in cases of high-grade sarcomas. In consistent with the literature, our study supports the notion that the likelihood of invasion increases as tumor size increases.

There was no statistically significant difference in the distribution of localization (femur or tibia) between invasive and noninvasive groups (p = 0.364).

In our study, intra-articular bone destruction (sensitivity 84%) and capsular invasion (sensitivity 92%) were identified as specific indicators of tumor invasion. Intra-articular bone destruction and capsular invasion were found to be statistically significant, similar to the studies by Bodden et al. [9]. The studies by Shima, and Ozaki reported that the specificity of intra-articular bone destruction was low [6,12]. Another statistically significant finding was the 84% specificity for intra-articular cartilage destruction. Intra-articular bone and cartilage destruction was assessed using T1 and fat-suppressed T2 or STIR.

Bodden et al. described intrasynovial tumor tissue visualization as a specific finding in the diagnosis of invasion [9]. In our study, the specificity of the presence of an intrasynovial tumor was 76%, and the sensitivity was 58%. Compared to other studies, the sensitivity for the presence of an intrasynovial tumor in our case series is low. We believe this is due to two factors: It has been reported that tumors located in the depths of the suprapatellar pouch may be mistakenly considered intra-articular [16]. Another reason is that most of the false positive diagnoses are extraosseous tumor extensions without capsular penetration, which are accepted as intra-articular tumors [14].

The indirect invasion findings of effusion, synovial contrast enhancement, and epiphyseal invasion were defined as less diagnostic and specific. In the studies presented in the literature, different results have been reported about whether joint effusion and synovial contrast enhancement are significant in the diagnosis of joint invasion [6,12]. In this study, effusion and synovial contrast enhancement were found to have low specificity and sensitivity in the diagnosis of invasion. These reactive changes may be caused by hyperemia, capsular irritation, or mechanical stress without invasion. Schima et al. reported that joint effusion alone has a positive predictive value of only 27% for intra-articular tumors, but a negative predictive value of 92% for tumor invasion [6]. Therefore, it suggests that the presence or absence of effusion is not a reliable marker for the diagnosis of joint invasion.

In contrast to the studies by Shima et al. and Bodden et al., the specificity and sensitivity of epiphyseal signal changes in the diagnosis of invasion were not significant in our study [6,9]. Infiltrative signal changes in the epiphyseal area have limited value in the diagnosis of joint invasion and should be evaluated together with other direct invasion findings.

### 4.1. Strengths and limitations

A key strength of our study lies in its focus on histopathological confirmation of joint invasion in all cases, providing robust validation of imaging findings. By limiting the analysis to knee osteosarcomas, our results offer specificity not found in broader studies covering multiple joints and tumor types. Compared to previous studies, our larger case series further enhances the reliability of our findings.

However, the retrospective design and single-center setting limit generalizability. In our study, imaging findings were evaluated by two radiologists and an orthopedist experienced in tumor surgery. The readers were blinded to histopathologic findings, and the final results were determined through a consensus decision. Therefore, interobserver agreement analysis was not performed, as the study focused on a collective decision-making process rather than independent evaluations by individual raters. With the wide range of sensitivity (56% – 92%) and specificity (48%–84%), we would like to emphasize that the primary aim of our study was to evaluate the consensus findings validated against histopathologic results as the reference standard. Despite this variability, the consensus-based approach provides a reliable assessment of joint invasion, supported by histopathologic validation, which strengthens the study’s conclusions.

To validate our findings, future prospective multicenter studies utilizing uniformly applied standardized imaging protocols across institutions are warranted.

In conclusion, this study underscores the diagnostic value of capsular infiltration and intra-articular bone destruction as direct MRI indicators of joint invasion in knee osteosarcoma. Indirect markers, while supplementary, are less reliable. Accurate preoperative diagnosis of joint invasion is critical to guide surgical strategies, minimizing the risks of inadequate resection or overtreatment. Future research should aim to refine diagnostic criteria and explore advanced imaging techniques to improve specificity and sensitivity.

## Figures and Tables

**Table 1:** Relationship between pathologic invasion and MR findings.

	Invasion(-) (N = 25)	Invasion(+) (N = 25)	Total (N = 50)	p-value
**Intrasynovial tumor tissue**				**0.039** **^1^**
Likert 1	**6 (31.6%)**	**13 (68.4%)**	**19 (100.0%)**	
Likert 2	**0 (0.0%)**	**1 (100.0%)**	**1 (100.0%)**	
Likert 3	**0 (0.0%)**	**1 (100.0%)**	**1 (100.0%)**	
Likert 4	**1 (50.0%)**	**1 (50.0%)**	**2 (100.0%)**	
Likert 5	**18 (66.7%)**	**9 (33.3%)**	**27 (100.0%)**	
**Intra-articular destruction of bone**				**0.029** **^1^**
Likert 1	**11 (35.5%)**	**20 (64.5%)**	**31 (100.0%)**	
Likert 2	**2 (66.7%)**	**1 (33.3%)**	**3 (100.0%)**	
Likert 3	**0 (0.0%)**	**0 (0.0%)**	**0 (0.0%)**	
Likert 4	**1 (100.0%)**	**0 (0.0%)**	**1 (100.0%)**	
Likert 5	**11 (73.3%)**	**4 (26.7%)**	**15 (100.0%)**	
**Intra-articular destruction of cartilage**				**0.015** **^1^**
Likert 1	**4 (23.5%)**	**13 (76.5%)**	**17 (100.0%)**	
Likert 2	**0 (0.0%)**	**1 (100.0%)**	**1 (100.0%)**	
Likert 3	**0 (0.0%)**	**0 (0.0%)**	**0 (0.0%)**	
Likert 4	**2 (66.7%)**	**1 (33.3%)**	**3 (100.0%)**	
Likert 5	**19 (65.5%)**	**10 (34.5%)**	**29 (100.0%)**	
**Capsular insertions invasion**				**0.012** **^1^**
Likert 1	**11 (36.7%)**	**19 (63.3%)**	**30 (100.0%)**	
Likert 2	**2 (40.0%)**	**3 (60.0%)**	**5 (100.0%)**	
Likert3	**0 (0.0%)**	**1 (100.0%)**	**1 (100.0%)**	
Likert 4	**2 (100.0%)**	**0 (0.0%)**	**2 (100.0%)**	
Likert 5	**10 (83.3%)**	**2 (16.7%)**	**12 (100.0%)**	
**Cruciate ligament invasion**				**0.005** **^1^**
Likert 1	**3 (17.6%)**	**14 (82.4%)**	**17 (100.0%)**	
Likert 2	**4 (66.7%)**	**2 (33.3%)**	**6 (100.0%)**	
Likert 3	**0 (0.0%)**	**0 (0.0%)**	**0 (0.0%)**	
Likert 4	**2 (50.0%)**	**2 (50.0%)**	**4 (100.0%)**	
Likert 5	**16 (69.6%)**	**7 (30.4%)**	**23 (100.0%)**	
**Epiphyseal bone marrow infiltration**				**0.235** **^1^**
Likert 1	**22 (46.8%)**	**25 (53.2%)**	**47 (100.0%)**	
Likert 2	**0 (0.0%)**	**0 (0.0%)**	**0 (0.0%)**	
Likert 3	**0 (0.0%)**	**0 (0.0%)**	**0 (0.0%)**	
Likert 4	**1 (100.0%)**	**0 (0.0%)**	**1 (100.0%)**	
Likert 5	**2 (100.0%)**	**0 (0.0%)**	**2 (100.0%)**	
**Epiphyseal edema signal intensity**				**0.110** **^1^**
Likert 1	**21 (45.7%)**	**25 (54.3%)**	**46 (100.0%)**	
Likert 2	**0 (0.0%)**	**0 (0.0%)**	**0 (0.0%)**	
Likert 3	**0 (0.0%)**	**0 (0.0%)**	**0 (0.0%)**	
Likert 4	**1 (100.0%)**	**0 (0.0%)**	**1 (100.0%)**	
Likert 5	**3 (100.0%)**	**0 (0.0%)**	**3 (100.0%)**	

Fisher’s Exact Test for Count Data

**Table 2:** Sensitivity and specificity values of MRI invasion findings.

	Sensitivity (%)	Specificity (%)	PPV (%)	NPV (%)
Intrasynovial tumour tissue	58 (14/24, 95%CI [37%–78%])	76 (19/25, 95%CI [55%–91%])	70 (0.46–0.88)	66 (0.46–0.82)
Destruction of intra-articular cartilage	56 (14/25, 95%CI [35%–76%])	84 (21/25, 95%CI [64%–95%])	78 (0.52–0.94)	66 (0.47–0.81)
Destruction of bone	84 (21/25, 95%CI [64%–95%])	48 (12/25, 95%CI [28%–69%])	62 (0.44–0.78)	75 (0.48–0.93)
Capsular insertion invasion	92 (22/24, 95%CI [73%–99%])	48 (12/25, 95%CI [28%–69%])	63 (0.45–0.79)	86 (0.57–0.98)
Cruciate ligament invasion	64 (16/25, 95%CI [43%–82%])	72 (18/25, 95%CI [% 51–% 88])	70 (0.47–0.87)	67 (0.46–0.83)
Epiphyseal bone marrow infiltration	100 (25/25, 95%CI [86%–100%])	12 (3/25, 95%CI [0.3%–31%])	53 (0.38–0.68)	100 (0.29–1.00)
Epiphyseal edema signal intensity	100 (25/25, 95%CI [86%–100%])	16 (4/25, 95%CI [0.5%–36%])	54 (0.39–0.69)	100 (0.40–1.00)
Synovial thickening and contrast enhancement	76 (19/25, 95% CI [55%–91%])	58 (14/24 95%CI [37%–78%])	66 (0.46–0.82)	70 (0.46–0.88)
Joint effusion	68 (17/25, 95%CI [46%–85%])	64 (16/25, 95%CI [43%–82%])	65 (0.44–0.83)	67 (0.45–0.84)

NPV, Negative predictive value; PPV, Positive predictive value; CI, Confidence interval.
